# Online Calibration of Polytomous Items Under the Graded Response Model

**DOI:** 10.3389/fpsyg.2019.03085

**Published:** 2020-01-23

**Authors:** Jianhua Xiong, Shuliang Ding, Fen Luo, Zhaosheng Luo

**Affiliations:** ^1^School of Psychology, Jiangxi Normal University, Nanchang, China; ^2^School of Computer and Information Engineering, Jiangxi Normal University, Nanchang, China

**Keywords:** online calibration, computerized adaptive testing, graded response model, squeezing average method, one EM cycle method, multiple EM cycle method

## Abstract

Computerized adaptive testing (CAT) is an efficient testing mode, which allows each examinee to answer appropriate items according his or her latent trait level. The implementation of CAT requires a large-scale item pool, and item pool needs to be frequently replenished with new items to ensure test validity and security. Online calibration is a technique to calibrate the parameters of new items in CAT, which seeds new items in the process of answering operational items, and estimates the parameters of new items through the response data of examinees on new items. The most popular estimation methods include one EM cycle method (OEM) and multiple EM cycle method (MEM) under dichotomous item response theory models. This paper extends OEM and MEM to the graded response model (GRM), a popular model for polytomous data with ordered categories. Two simulation studies were carried out to explore online calibration under a variety of conditions, including calibration design, initial item parameter calculation methods, calibration methods, calibration sample size and the number of categories. Results show that the calibration accuracy of new items were acceptable, and which were affected by the interaction of some factors, therefore some conclusions were given.

## Introduction

Computerized adaptive testing (CAT), which is considered to be one of the most important applications of item response theory (IRT; Lord, 1980), is a tailored test mode (e.g., Chang and Zhang, 2002; Chang, 2015). The goal of CAT is to construct an optimal test for each examinee (Meijer and Nering, 1999). Compared with the traditional paper-pencil test (PandP), CAT has many advantages such as more flexible testing time, more diverse items, shorter test length, more accurate ability estimation, and more timely score reporting (e.g., Weiss, 1982; Meijer and Nering, 1999; Cheng and Chang, 2009; Wang and Chang, 2011; Wang et al., 2013). Therefore, many large-scale evaluation programs such as the Graduate Management Admission Test (GMAT) and the Armed Services Vocational Aptitude Battery (ASVAB; Sands et al., 1997) adopted the CAT test mode (Chang and Ying, 2009).

The implementation of CAT requires a large-scale item pool, and the maintenance and management of item pool is critical to ensure the validity and security of CAT. After a period of time, some operational items may be no longer suitable for use due to overexposure, obsoleteness, or flaw, thus it is necessary to replace unsuitable items by new ones (Wainer and Mislevy, 1990; Zheng, 2014; Zheng and Chang, 2017). The new items should be precisely calibrated before being put into the item pool for use formally. Moreover, the calibration accuracy of the new items has great influence on the estimation accuracy of the examinees' latent trait in the ensuing CAT sessions (e.g., van der Linden and Glas, 2000; Chang and Lu, 2010).

Wainer and Mislevy (1990) proposed two strategies for calibrating new items based on CAT in the literature. The first strategy is traditional offline calibration with anchor-item design. Namely, set some anchor items between the new and the operational items, and do equating transformation through the collected responses to ensure the item parameters of the new items and those of the operational items on the same scale. Because the traditional calibration method needs to organize P and P test in advance, there are some shortages, such as the consumption of manpower and material resources, the easy exposure of new items and so on. The second strategy is online calibration, which refers to the process of assigning the new items to examinees during the course of their adaptive tests and then estimating the item parameters of new items based on the collected responses. In the online calibration framework, new items can be embedded inconspicuously within the operational tests, and be pretested and calibrated in the same testing environment as the operational items. Compared with the traditional calibration, online calibration is not only time-saving but also cost-effective. It places new items on the same scale as the operational items without *post hoc* scaling.

Online calibration design and online calibration method are two crucial aspects of online calibration (Chen and Xin, 2014). Online calibration design refers to the way which the new items are assigned to examinees during the CAT process, and collects the responses of the new items. Online calibration design mainly includes two types. One is random design, and the other is adaptive design. Random design randomly selects a new item and then stochastically seeds it in the current examinee's adaptive test (Wainer and Mislevy, 1990). Adaptive design selects the most suitable new item according to some criterion when he or she reaches a seeding location (He and Chen, 2019). The online calibration method uses the responses collected during the online calibration design phase to estimate the item parameters of new items. The most popular estimation methods proposed for online calibration include one EM cycle method (OEM; Wainer and Mislevy, 1990) and multiple EM cycle method (MEM; Ban et al., 2001).

There are many studies on online calibration based on dichotomously scored models (e.g., You et al., 2010; Chen et al., 2012; van der Linden and Ren, 2015; He et al., 2017, 2019). One purpose of modern item response theory research is to exhaust all types of models to cover test data from any “natural” form (van der Linden and Hambleton, 1997). And compared with dichotomously scored items, polytomously scored items have many advantages, such as measuring more complex knowledge structure and providing higher item and test information. Therefore, examinees' ability can be estimated with greater precision by the same number of items, or the same level of precision can be obtained with fewer items. More and more tests involving polytomously scored items have emerged. However, online calibration of polytomously scored model is reported rarely. Zheng (2016) extends the formula, procedure and algorithm of online calibration under dichotomously scored models to the generalized partial credit model (GPCM). The extended formulas and algorithms are studied by simulation method, and some constructive conclusions are obtained. The graded response model (GRM; Samejima, 1969, 1996), like GPCM, is a polytomously scored model. But they have many differences. First, the ideas of model construction are different, GPCM is a division model, that is, the proportion of part to whole. In contrast, GRM is a deviation model, that is, the difference between adjacent categories. Second, the meanings of difficulty parameters in GRM and GPCM models are different, GPCM emphasizes the difficulty of each step on an item, and the difficulty value does not necessarily increase monotonously, GRM emphasizes the difficulty of getting different scores on an item, and the difficulty value increases monotonously. Therefore, it is necessary to discuss online calibration based on GRM, it is of great significance to the expansion of the item pool with GRM items.

The structure of this article is as follows. First, the GRM, an IRT model used in this research is introduced. Second, online calibration method (OEM and MEM method) based on GRM is introduced. Two methods for calculating initial item parameters are given in detail. Third, two simulation studies are designed, and the research results are presented. Fourth, a batch of real data are used to verify the validity of the method. The last part involves conclusions, a supplementary study, discussions, and directions for future research.

## Methodology

### The GRM

The GRM is an IRT model suitable for polytomous data with ordered categories. It is an extension of two parameters logistic model (2PLM). In GRM, an examinee's likelihood of responding in a particular response category is obtained by two steps. First, category boundary response functions (CBRFs) are calculated to determine boundary decision probabilities of *t* response categories for each item. The equation for a CBRF is similar to 2PLM for dichotomous data:

(1) $$\begin{array}{l} p_{ijt}^{*}=\frac{1}{1+\text{ exp}\left(-D\cdot a_{j}\left(\theta_{i}-b_{jt}\right)\right)} \end{array}$$

In Equation (1), $p_{ijt}^{*}$ is the probability that an examinee with ability level θ_*i*_ will respond positively at the boundary of category *t* for item *j* where *t* = 1, 2, ⋯*f*_*j*_, θ_*i*_ represents the *i*th examinee's ability; *a*_*j*_ represents the item discrimination parameter or slope for item *j*; *b*_*jt*_ represents the item difficulty parameter or category location. Importantly, the values of *b*_*jt*_ should satisfy monotonically increasing, that is *b*_*j*1_ < *b*_*j*2_ < ⋯*b*_*jt*_ < ⋯*b*_*j*,_*f*__*j*__.

In the second step of GRM, the probability of responding in a particular category is determined by CBRF, which are derived by subtracting $p_{ijt}^{*}$ from the following category. The process is illustrated in Equation (2) (adapted from Embretson and Reise, 2000).

(2) $$\begin{array}{l} p_{ijt}=p_{ijt}^{*}-p_{ij,t+1}^{*} \end{array}$$

Further, make the following constraints, $p_{ij0}^{*}=1$, namely, the probability of scoring more than 0 must be 1; $P_{ij,fj+1}^{*}=0$, that is, the probability of scoring more than the item's full score is naturally 0.

### Extend OEM and MEM Methods to GRM

Under the dichotomous model, OEM (Wainer and Mislevy, 1990) and MEM (Ban et al., 2001) are based on the framework of MMLE with the EM algorithm. Their main difference is the number of EM cycles. The OEM method takes just one E step using the posterior distribution of ability, which is estimated based on item responses only from the operational CAT items, and just one M step to estimate the new item parameters, involving response data from only the new items. The MEM method is similar mathematically to the OEM method. The first EM cycle of the MEM method is the same as the OEM method. The parameter estimates of new items obtained from the first EM cycle is regarded as the initial values of the new items for the second EM cycle. However, from the second E step, the MEM method uses item responses on both the operational items and new items to obtain the posterior distribution. For each M step iteration, the item parameter estimates for the operational items are fixed, whereas parameter estimates for the new items are updated until the new item parameter estimates converge. The principles of OEM and MEM under GRM are basically the same as those under the dichotomous model, but there are some differences in implementation details. The details of OEM and MEM implementation under GRM are described below.

#### OEM

OEM has only one EM cycle. For each examinee *i* = 1, 2, ⋯*N*_*j*_ who takes item *j*, *q*_*i*_ denotes his/her responses to the operational items, η_*op*_ is a vector of the known item parameters of the operational items. The E-step of the OEM method marginalizes the log-likelihood of new item *j* using *q*_*i*_ and η_*op*_. Based on the common assumption that examinees are independent from each other, the log-likelihood of item *j* from the *N*_*j*_ examinees are summed up as the final marginalized log-likelihood of item *j* to be taken to the subsequent M-step. The M-step seeks the item parameter vector ${\hat{\eta }}_{j}$ that maximizes the final marginalized log-likelihood of item *j*.

These two steps are adapted from described in Muraki (1990) of item parameter estimation. The difference between the algorithms here for online calibration and Muraki's algorithm is in the computation of the two quantities: ${\bar{r}}_{jtk}$ and ${\bar{f}}_{k}$, where ${\bar{r}}_{jtk}$ is the temporary expected frequency of the *t*th category response of item *j* at the *k*th quadrature point; ${\bar{f}}_{k}$ is the temporary expected sample size at quadrature point *k*. In his original EM algorithm, every examinee receives the same set of items. In the online calibration setting, as described earlier in this article, each new item *j* is administered to a different sample of examinees; and each examinee who takes new item *j* takes a different set of operational items. To adapt these variations, the formulae for ${\bar{r}}_{jtk}$ and ${\bar{f}}_{k}$ in the EM algorithm are modified into as follows:

(3) $$\begin{array}{l} {\bar{r}}_{jtk}=\overset{N_{j}}{\underset{i=1}{\sum }}u_{ijt}h\left(X_{k}\right) \end{array}$$

(4) $$\begin{array}{l} {\bar{f}}_{k}=\overset{N_{j}}{\underset{i=1}{\sum }}h\left(X_{k}\right) \end{array}$$

(5) $$\begin{array}{l} h\left(X_{k}\right)=\frac{L_{i}\left(X_{k}\right)A\left(X_{k}\right)}{\overset{K}{\underset{k=1}{\sum }}L_{i}\left(X_{k}\right)A\left(X_{k}\right)} \end{array}$$

(6) $$\begin{array}{l} L_{i}(X_{k})=\prod_{h=1}^{m_{i}}\prod_{t=1}^{f_{h}}\left[p_{ht}(X_{k}\right){]}^{q_{iht}} \end{array}$$

Where *i* = 1, 2, ⋯ , *N*_*j*_ denote the *N*_*j*_ examinees who received new item *j*; *X*_*k*_ is the quadrature point; *A*(*X*_*k*_) is the corresponding weight, which is approximately the standard normal probability density at the point *X*_*k*_, assuming there are *K* quadrature points, such that $\sum_{k=1}^{K}A\left(X_{k}\right)=1$.*U*_*ijt*_ is an indicator variable expressed in a binary format; *U*_*ijt*_ = 1 represents examinee *i* scored exactly *t* on new item *j*; otherwise *U*_*ijt*_ = 0.*L*_*i*_(*X*_*k*_) is the likelihood of examinee *i*'s response to all operational items given quadrature point *X*_*k*_; *h* denotes the *h*th operational items answered by examinee *i*; *f*_*h*_ is the number of categories of *h*th operational item, *p*_*ht*_(*X*_*k*_) is the probability of correct response to the *t*th category of item *h* at given quadrature point *X*_*k*_, *q*_*iht*_ is an indicator variable too, which denotes the examinee *i*'s responses to operational item *h* in a binary format to category *t*.

With the one EM cycle in the OEM method, the revised ${\bar{r}}_{jtk}$ and ${\bar{f}}_{k}$ are inserted into the Newton-Raphon iteration in the single EM cycle to get a set of parameter estimates.

#### MEM

The MEM method allows multiple EM cycles. The first cycle is the same as OEM. Beginning with the second cycle, response data from both the operational items and the new items are used to update the posterior ability distribution in the E-step. Specifically, the only change in computation from OEM is that beginning with the second cycle of MEM, *L*_*i*_(*X*_*k*_) is replaced by:

(7) $$\begin{array}{l} L_{i}(X_{k})=\left(\prod_{h=1}^{m_{i}}\prod_{t=1}^{f_{h}}\left[p_{ht}(X_{k}\right){]}^{q_{iht}}\right)\left(\prod_{t=1}^{f_{j}}{\left[p_{jt}(X_{k})\right]}^{x_{ijt}}\right) \end{array}$$

Where *x*_*ijt*_ denotes examinee *i*'s response to new item *j* in the binary format for category *t*.

The E-step and the M-step iterate until a certain convergence criterion is met, for example the maximum absolute change in the item parameters between two consecutive EM cycles are less than a small threshold.

### Calculate the Initial Value of OEM and MEM

OEM and MEM are both iterative algorithms, the initial item parameters have a great influence on the calibration accuracy. However, there are few reports on the calculation of initial iteration values. In the dichotomous model, a squeezing average method is given to compute the initial value of difficulty parameter and a biserial correlation method is used to compute the initial value of discrimination parameter (You et al., 2010). Under GRM, Xiong et al. (2018) also proposed a methods for calculating the initial item parameters, namely, deleting extremum and squeezing average method and polyserial correlation coefficient method. They had better calibration results under the experimental conditions given in these literatures (You et al., 2010; Xiong et al., 2018). Their theories and implementation details are as follows.

#### Deleting Extremum and Squeezing Average Method

Under the dichotomous model, according to the characteristics of the item response curve, the correctness of the examinee's response to a certain item is related with the ratio of his/her ability to the difficulty parameter of the item. When the ratio is more than 1, the correct response probability is high; otherwise, the correct response probability is low. For the one-parameter logistic model (1PLM), when the examinee's ability value is equal to the difficulty of one item, his/her correct response probability on the item is 0.5. Therefore, as long as the number of responses is sufficiently large for one item, there must be some examinees whose abilities approach to the difficulty parameter of the item (You et al., 2010), and the abilities of these examinees can be used to estimate the difficulty parameter of the item. The method is called “squeezing average method.” Under GRM, for a certain item, the difficulty of getting a high score is higher than that of getting a low score, so the initial parameters of different category can be squeezed out by the ability of the examinees who get the adjacent scores.

The steps of the squeezing average method (You et al., 2010) are described as follows. At first, put the ability values of all examinees who answered correctly on item *j* into the set correct(*j*), then sort correct(*j*) in ascending order; and put the ability values of all examinees who answered incorrectly on item *j* into the set wrong(*j*), then sort wrong(*j*) in descending order. Second, use the low part of correct(*j*) and the high part of wrong(*j*) to squeeze the difficulty of the item *j*. Because not all examinees' abilities in correct(*j*) or wrong(*j*) are used for squeezing, it is worth exploring how many examinees' abilities are used to squeeze item difficulty parameter. An empirical value of 18 is suggested by You et al. (2010).

Under the GRM model, GRM has multiple difficulty parameters, so multiple squeezing processes are required. For example, for the initial difficulty parameter of the *t*th category of the new item *j*, the ability of the examinees who scored *t* and *t*+1 on the item are used to squeeze. Pilot studies have shown that the result is unstable if the sample size for squeezing is still set to 18. A more flexible range of sample size for squeezing method, named “deleting extremum and squeezing average method,” is proposed based on the original squeezing average method (Xiong et al., 2018). The ability of examinees who got *t* score in item *j* are put into one set, there are *f*_*j*_ sets for item *j*, and each set is sorted in ascending order by ability value. Then the top 5% and the bottom 5% of each set are deleted. The “deleting extremum and squeezing average method” can be formally expressed as:

(8) $$\begin{array}{rcl} {\text{b}}_{\text{jt}}=\{(mean\left(\overset{c\left(j,t\right)\text{*}95\;\%\;}{\underset{i=c\left(j,t\right)\text{*}5\;\%\;}{\sum }}cap\left(t,i,j\right)\right)\\ \; & \; & +mean\left(\overset{c\left(j,t+1\right)*95\%}{\underset{i=c\left(j,t+1\right)*5\%}{\sum }}cap\left(t+1,i,j\right)\right))/2 \end{array}$$

Where *cap*(*t, i, j*) is the ability of the *i*th examinee's who got *t* score on item *j*, *c*(*j, t*) is the number of examinees who scored *t* on item *j*, *cap*(*t* + 1, *i, j*) and *c*(*j, t* + 1) have the similar meaning.

In actual life, the evaluation of a contestant is generally based on a set of scores given by the experts. The highest and lowest score are removed, and then the average is taken, deleting extremum and squeezing average method takes this idea. The practice of choosing 5% as the extreme value in Equation (8) is derived from the way to obtain the initial value of the guess parameter under the three-parameter logistic model (3PLM). Pilot study also showed that the value had better results. It's easy to implement and guarantee the accuracy of parameter estimation.

#### Polyserial Correlation Coefficient Method

The polyserial correlation coefficient method is a common statistical method (Olsson et al., 1982), which is used to initialize the discrimination parameter and difficulty parameter of new items based on the examinee's responses. This method can be depicted by the following steps:

**Step 1:** For each new item, the pass rate of each category is calculated by using the responses of the examinees to the item, that is, $P_{jt}^{*}=n_{jt}/N$, where *N* is the total number of examinees, and *n*_*jt*_ is the number of examinees whose scores on the new item *j* are not lower than *t*.
**Step 2:** Convert $P_{jt}^{*}$ to standard normal fraction *Z*_*jt*_; then calculate the corresponding normal density function value *h*(*Z*_*jt*_). The specific calculation formula is as follows:
  (9) $$\begin{array}{l} y_{j}=-\text{ ln}\left(4P_{jt}^{*}\left(1-P_{jt}^{*}\right)\right) \end{array}$$
  (10) $$\begin{array}{l} Z_{jt}=sign\left(P_{jt}^{*}-\frac{1}{2}\right)\sqrt{y_{j}\left(2.0611786-\frac{5.7262204}{y_{j}+11.640595}\right)} \end{array}$$
  (11) $$\begin{array}{l} h\left(Z_{jt}\right)=\frac{1}{\sqrt{2\pi }}\text{exp}\left(-\frac{1}{2}Z_{jt}^{2}\right) \end{array}$$
**Step 3:** Calculate the standard deviation (σ_*j*_) of the score on the new item *j*, and the correlation coefficient (*r*_*j*_) between the score of the new item *j* and the total score; then the point polyserial correlation coefficient is obtained via the following equation:
  (12) $$\begin{array}{l} rpp_{j}=r_{j}*\sigma_{j}/\overset{f_{j}}{\underset{t=1}{\sum }}h\left(Z_{jt}\right) \end{array}$$
**Step 4:** Transform the point polyserial correlation coefficient into polyserial correlation coefficient, that is:
  (13) $$\begin{array}{l} rp_{j}=rpp_{j}*\sigma_{j}/\overset{f_{j}}{\underset{t=1}{\sum }}h\left(Z_{jt}\right) \end{array}$$
**Step 5:** Calculate the initial value of the discrimination and difficulty of the new item *j*; the formula is:
  (14) $$\begin{array}{l} a_{j}=rp_{j}/\sqrt{1-rp_{j}^{2}}\;b_{jt}=-Z_{j,t-1}/rp_{j} \end{array}$$

Two methods of calculating the initial parameters of new items are given. The first method is called polyserial-initial method, abbreviated as Poly-Ini method, with this method, both *a*-parameter and *b*-parameters are calculated by polyserial correlation coefficient; the second method is called polyserial-squeezing-initial method, abbreviated as Poly-Sq-Ini method, with this method, *a*-parameter is calculated by polyserial correlation coefficient method and *b*-parameters are obtained by deleting extremum and squeezing average method.

## Simulation Study

### Research Objectives

Two simulation studies were conducted using programs written in Python 3.7. The program simulated the entire calibration workflow including the implementation of CAT and the calibration of the new items, and replicated 100 times in each circumstance. The main purpose of Study 1 is to explore the calibration results under a set of conditions fully crossed by two online calibration design methods (random design, adaptive design), two initial item parameter calculation methods (Poly-Ini method, Poly-Sq-Ini method), two calibration methods (OEM, MEM). There are 8 combinations, each combination takes 3-categories as an example.

The main purpose of Study 2 is to explore the calibration results under different calibration sample size and different number of categories. Two factors were manipulated: calibration sample size (300, 400, 500, 600, and 700) and the number of categories of new items (2, 3, 4, and 5). There are 20 combinations. Random design, Poly-Sq-Ini method and MEM are adopted in each combinations.

### Generation of Items and Examinees

Suppose there are 1000 operational items with various categories (2–5 categories) in the CAT item pool, item parameters were randomly generated under GRM from the following distributions:

$$\begin{array}{l} a_{j}\;\sim \text{ log }normal\left(0,1\right),\;b_{jt}\;\sim \;normal\left(0,1\right) \end{array}$$

*j* = 1, 2, ⋯1000, *t* = 1, 2, ⋯*f*_*j*_,*f*_*j*_ is the number of categories. In addition, the generated *a*-parameter was truncated between 0.2 and 2.5, *b*-parameter was truncated between −3 and 3, and *b*_*j*1_ < *b*_*j*2_ < ⋯*b*_*jt*_ < ⋯*b*_*j*,_*f*__*j*__ in this paper.

A total number of 20 new items were generated in the same manner with the operational items.

3,000 examinees' ability values (θ) were randomly drawn from the standard normal distribution θ ~ *normal*(0,1), and θ was truncated between −3 and 3 too.

### Simulation Details

The CAT test length is fixed 25 items, including 20 operational items and 5 new items. During the CAT test, the maximum Fisher information method (MFI; Lord, 1980) was chosen as the operational item selection method for its advantage of high accuracy. The Fisher information of an examinee *i* on a GRM item *j* was formulated as below:

(15) $$\begin{array}{l} I_{j}\left(\theta_{i}\right)=a_{j}^{2}\overset{f_{j}}{\underset{t=1}{\sum }}p_{ijt}{\left(1-p_{ijt}^{*}-p_{ij,t+1}^{*}\right)}^{2} \end{array}$$

During operational item selection, provisional θ estimates were used to replace the θ's in the formulae. After each operational item is administered, the examinee ability parameter $\hat{\theta }$ was updated by expected a posteriori (EAP) method (Baker and Kim, 2004).

The number of examinees who answer each new item must be sufficiently large to provide accurate item parameter estimates without placing an undue burden on examinees (Wainer and Mislevy, 1990). This paper investigates one sample size (3,000) and assumes that each examinee answers 5 new items, thus the number of examinees who answer each new item is approximately 750 [(3,000 × 5)/20] on average as in previous studies (e.g., Chen et al., 2012; Chen and Wang, 2016; He et al., 2017). In Study 1, the number of examinees to each new item is set 700. In addition, calibration accuracy may be affected by the calibration samples per new item. In Study 2, the number of examinees to each new item is set as 300, 400, 500, 600, 700.

In study 1, random design and adaptive design are considered. There are some researches adopted random design to assign the new items to the examinees during CAT due to its convenient implementation and acceptable calibration precision (e.g., Wainer and Mislevy, 1990; Ban et al., 2001; Chen et al., 2012; He et al., 2017). And match-b selection method (MATB) is selected for adaptive design in this study, which matches the mean of *b*-parameters with the provisional $\hat{\theta }$ of examinee (Zheng, 2016). Every time an examinee reaches a seeding location, the distance between his or her current $\hat{\theta }$ and the mean of provisional *b*-parameters was computed for each new item, and the item with the shortest absolute distance was selected. In order to obtain the initial parameter of new items, this study uses a data-based method, that is, the new items are first randomly assigned to a sub-group of examinees and are pre-estimated item parameters, then for the remaining examinees, these new items are selected adaptively according to their initial parameters to fit the examinees' current ability. The item parameters of each new item are updated each time they receive a fixed number of new responses (van der Linden and Ren, 2015; Zheng, 2016; He et al., 2019), in this study, the fixed number of new responses was set 20. The proportion of the sample size used in two different phases was specified as 1:1 in this study.

### Evaluation Criteria

The calibration accuracy of the new items was evaluated by root mean square error (RMSE) and bias. They quantify the recovery between the estimated and true parameter values, and the calculation formulas based on vector are as follows (He and Chen, 2019; He et al., 2019):

(16) $$\begin{array}{l} RMSE_{x}=\sqrt{\left(\overset{R}{\underset{r=1}{\sum }}\overset{M}{\underset{j=1}{\sum }}{\left({\hat{x}}_{j}^{\left(r\right)}-x_{j}^{\left(r\right)}\right)}^{2}\right)/\left(R\times M\right)} \end{array}$$

(17) $$\begin{array}{l} bias_{x}=\left(\overset{R}{\underset{r=1}{\sum }}\overset{M}{\underset{j=1}{\sum }}\left({\hat{x}}_{j}^{\left(r\right)}-x_{j}^{\left(r\right)}\right)\right)/\left(R\times M\right) \end{array}$$

Where *x* denotes the specific element in the item parameter vector, such as *a*-parameter, *b*_*f*_*j*__-parameters, *R* and *M* denotes replications and the number of new items respectively.

In order to evaluate the overall recovery of *b*-parameters under different categories, the average RMSE and bias of *b*-parameters, named mean(b), are defined as follows:

(18) $$\begin{array}{l} RMSE_{mean\left(b\right)}=\sqrt{\left(\overset{R}{\underset{r=1}{\sum }}\overset{M}{\underset{j=1}{\sum }}\overset{f_{j}}{\underset{t}{\sum }}{\left({\hat{b}}_{jt}^{\left(r\right)}-b_{jt}^{\left(r\right)}\right)}^{2}\right)/\left(R\times M\times f_{j}\right)} \end{array}$$

(19) $$\begin{array}{l} bias_{mean\left(b\right)}=\left(\overset{R}{\underset{r=1}{\sum }}\overset{M}{\underset{j=1}{\sum }}\overset{f_{j}}{\underset{t}{\sum }}\left({\hat{b}}_{jt}^{\left(r\right)}-b_{jt}^{\left(r\right)}\right)\right)/\left(R\times M\times f_{j}\right) \end{array}$$

Smaller RMSE indicates higher calibration precision. If bias is close to 0, the calibration could be regarded as unbiased.

### Results and Conclusion

#### Study 1

**Figure 1:**
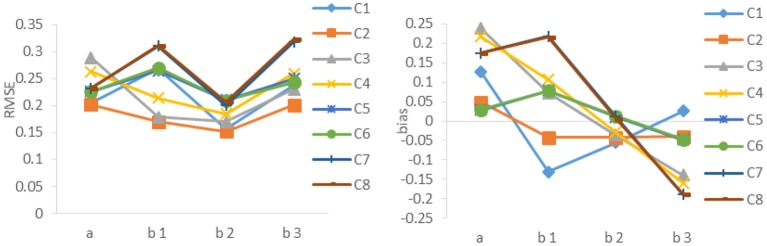
RMSE and bias of *a*- parameter and *b*-parameters under different combinations. C1 denotes the combination of Random, Poly-Sq-Ini and OEM; C2 denotes the combination of Random, Poly-Sq-Ini and MEM; C3 denotes the combination of Random, Poly-Ini and OEM; C4 denotes the combination of Random, Poly-Ini and MEM; C5 denotes the combination of Adaptive, Poly-Sq-Ini and OEM; C6 denotes the combination of Adaptive, Poly-Sq-Ini and MEM; C7 denotes the combination of Adaptive, Poly-Ini and OEM; C8 denotes the combination of Adaptive, Poly-Ini and MEM.

The results of Study 1 are shown in Tables 1, 2 and Figure 1, using two separate criteria (RMSE and bias) to evaluate the calibration results under different combinations. As can be seen from Tables 1, 2 and Figure 1, (1) the RMSE values obtained by the combination of random design, Poly-Sq-Ini method and MEM (the combination denoted by C2) were the smallest, and the bias obtained by C2 also had better performance, although not always the best. Which provided the basis for the simulation design of Study 2. (2) The calculation of initial item parameters had a great influence on the calibration results, Poly-Sq-Ini method had better performance under most experimental combinations, the bias had the same trend as RMSE, which showed that the Poly-Sq-Ini method is a feasible method. (3) Comparing OEM and MEM, when adaptive design was adopted, OEM and MEM generated quite comparable RMSE and bias values, when random design was adopted, there are two aspects, MEM was more accurate than OEM if Poly-Sq-Ini method was adopted to compute initial item parameters, otherwise OEM was more accurate than MEM. (4) Comparing random design and adaptive design, the RMSE of *b*-parameters generated by random design were smaller than those by adaptive design, although the *a*-parameters generated by random design were not absolutely superior, the most accurate *a*-parameters still came from random design. The result seems counter-intuitive, one possible explanation for this result is that the simulated examinee's ability distribution is normal, random design leads to an approximately normal distribution of ability for each new item. For adaptive design, the distributions of ability received by each new item may be skewed (Zheng, 2016). The other possible explanation is that the proportion of the sample size used in random phase and adaptive phase would affect the calibration results (Chen et al., 2012).

**Table 1:** RMSE under different combinations.

**Calibration design**	**Method of calculating initial item parameters**	**Calibration method**	**RMSE**			
			***a***	***b1***	***b2***	***b3***
Random	Poly-Sq-Ini	OEM	0.2047	0.2696	0.1567	0.2377
		MEM	0.2022	0.1705	0.1522	0.2009
	Poly-Ini	OEM	0.2892	0.1789	0.1705	0.2306
		MEM	0.2632	0.2142	0.1847	0.2595
Adaptive	Poly-Sq-Ini	OEM	0.2266	0.2651	0.2108	0.2501
		MEM	0.2259	0.2700	0.2101	0.2433
	Poly-Ini	OEM	0.2324	0.3106	0.2005	0.3179
		MEM	0.2324	0.3116	0.2070	0.3231

**Table 2:** Bias under different combinations.

**Calibration design**	**Method of calculating initial item parameters**	**Calibration method**	**bias**			
			***a***	***b1***	***b2***	***b3***
Random	Poly-Sq-Ini	OEM	0.1258	−0.1310	−0.0549	0.0261
		MEM	0.0483	−0.0423	−0.0422	−0.0398
	Poly-Ini	OEM	0.2380	0.0727	−0.0367	−0.1391
		MEM	0.2163	0.1065	−0.0292	−0.1589
Adaptive	Poly-Sq-Ini	OEM	0.0286	0.0777	0.0099	−0.0482
		MEM	0.0296	0.0783	0.0126	−0.0472
	Poly-Ini	OEM	0.1744	0.2182	0.0120	−0.1887
		MEM	0.1751	0.2159	0.0056	−0.1875

#### Study 2

**Figure 2:**
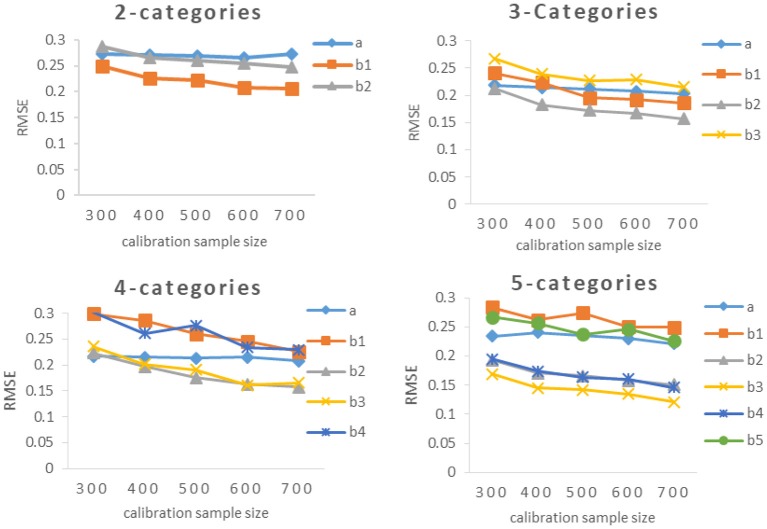
RMSE of *a*- parameter and *b*-parameters under different categories.

**Figure 3:**
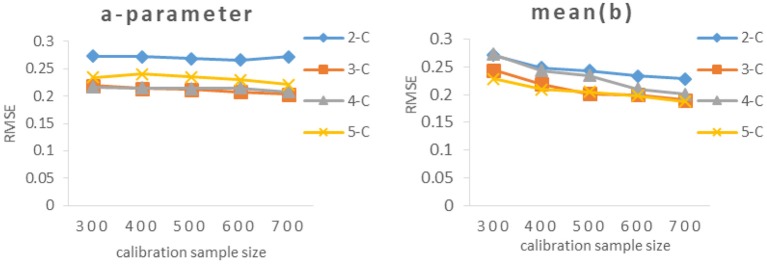
RMSE of different calibration sample size under different categories. 2-C, 2-categories; 3-C, 3-categories; 4-C, 4-categories; 5-C, 5-categories. Figure 5 also has the same definition.

**Figure 4:**
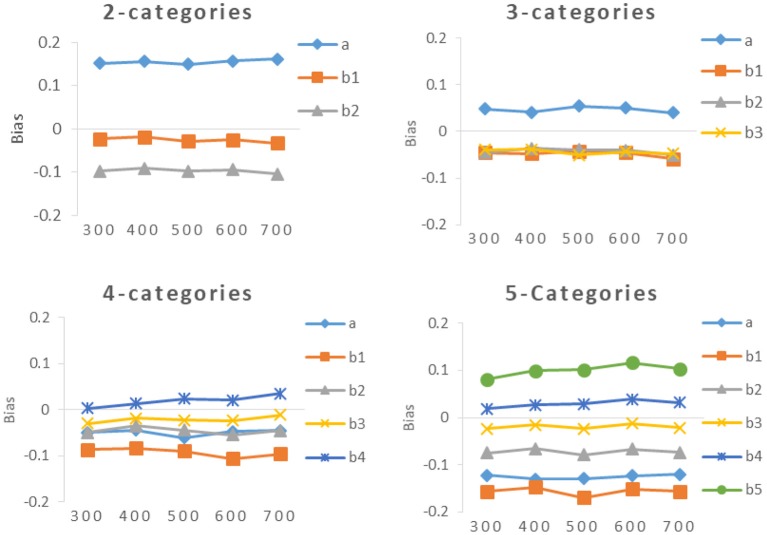
Bias of *a*- parameter and *b*-parameters under different categories.

**Figure 5:**
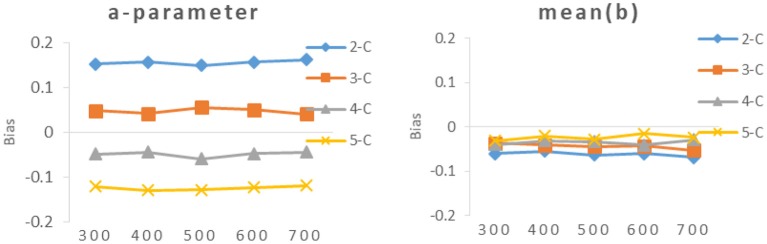
Bias of different calibration sample size under different categories.

The results of Study 2 are shown in Tables 3, 4 and Figures 2–**5**. As can be seen from Table 3 and Figure 2, under various categories, with the increase of calibration sample size, the RMSE of *b*-parameters were decreasing, but the decline extent was decreasing also. While the calibration sample size had little effect on the RMSE of *a*-parameters, even under 2-categories and 5-categories, the RMSE increases with the increase of sample size. In addition, it was an interesting observation, the RMSE of *b*-parameters under different category of the same item were different. In general, the RMSE of the middle category were smaller, while the RMSE of the beginning and ending category were larger. The possible explanation for this result is that the *b*-parameters in GRM were monotonically increasing, and most of the examinees' scores were concentrated on the middle category. Thus there were relatively few examinees with the lowest score and the highest score, and the sample size would affect the estimation accuracy of new items.

**Table 3:** RMSE of different calibration sample size under different categories.

**Categories**	**RMSE**	**Calibration sample size**				
		**300**	**400**	**500**	**600**	**700**
*f* = 2	*a*	0.2730	0.2716	0.2683	0.2656	0.2722
	*b_1_*	0.2495	0.2259	0.2216	0.2078	0.2060
	*b_2_*	0.2876	0.2660	0.2602	0.2554	0.2470
	Mean(*b*)	0.2706	0.2481	0.2427	0.2338	0.2286
*f =* 3	*a*	0.2189	0.2141	0.2119	0.2074	0.2033
	*b_1_*	0.2413	0.2237	0.1954	0.1919	0.1865
	*b_2_*	0.2127	0.1827	0.1723	0.1673	0.1568
	*b_3_*	0.2674	0.2395	0.2270	0.2249	0.2156
	Mean(*b*)	0.2439	0.2187	0.2014	0.1993	0.1899
*f* = 4	*a*	0.2166	0.2150	0.2138	0.2149	0.2081
	*b_1_*	0.2989	0.2866	0.2599	0.2458	0.2262
	*b_2_*	0.2232	0.1968	0.1760	0.1634	0.1577
	*b_3_*	0.2357	0.2016	0.1908	0.1610	0.1659
	*b_4_*	0.2996	0.2611	0.2564	0.2337	0.2294
	Mean(*b*)	0.2722	0.2432	0.2345	0.2098	0.2007
*f* = 5	*a*	0.2340	0.2407	0.2353	0.2301	0.2208
	*b_1_*	0.2837	0.2616	0.2604	0.2503	0.2491
	*b_2_*	0.1929	0.1706	0.1662	0.1583	0.1511
	*b_3_*	0.1693	0.1451	0.1419	0.1346	0.1210
	*b_4_*	0.1950	0.1743	0.1633	0.1600	0.1462
	*b_5_*	0.2672	0.2565	0.2368	0.2356	0.2257
	Mean(*b*)	0.2284	0.2095	0.2044	0.1976	0.1873

**Table 4:** Bias of different calibration sample size under different categories.

**Categories**	**Bias**	**Calibration sample size**				
		**300**	**400**	**500**	**600**	**700**
*f* = 2	*a*	0.1517	0.1561	0.1488	0.1564	0.1611
	*b_1_*	−0.0231	−0.0193	−0.0289	−0.0253	−0.0336
	*b_2_*	−0.0976	−0.0912	−0.0979	−0.0945	−0.1047
	Mean(*b*)	−0.0603	−0.0553	−0.0634	−0.0599	−0.0692
*f =* 3	*a*	0.0479	0.0415	0.0546	0.0500	0.0398
	*b_1_*	−0.0451	−0.0479	−0.0424	−0.0457	−0.0589
	*b_2_*	−0.046	−0.0365	−0.0395	−0.0403	−0.0502
	*b_3_*	−0.0398	−0.0385	−0.0502	−0.0435	−0.0478
	Mean(*b*)	−0.0365	−0.0409	−0.0440	−0.0432	−0.0523
*f* = 4	*a*	−0.0491	−0.0445	−0.0602	−0.0477	−0.0449
	*b_1_*	−0.086	−0.0829	−0.089	−0.1059	−0.0957
	*b_2_*	−0.0491	−0.0354	−0.0444	−0.0544	−0.0451
	*b_3_*	−0.0298	−0.0186	−0.0227	−0.0238	−0.0115
	*b_4_*	0.0032	0.0132	0.0239	0.0204	0.0347
	Mean(*b*)	−0.0404	−0.0309	−0.0330	−0.0409	−0.0294
*f* = 5	*a*	−0.1217	−0.1305	−0.1289	−0.1232	−0.1199
	*b_1_*	−0.1567	−0.1473	−0.1699	−0.1519	−0.1568
	*b_2_*	−0.0752	−0.0662	−0.0789	−0.0665	−0.0737
	*b_3_*	−0.0238	−0.0155	−0.0239	−0.0126	−0.0211
	*b_4_*	0.0192	0.0273	0.0294	0.0390	0.0315
	*b_5_*	0.0817	0.0990	0.1018	0.1168	0.1031
	Mean(*b*)	−0.0309	−0.0205	−0.0283	−0.0150	−0.0233

As can be seen from Table 3 and Figure 3, the RMSEs of *a*-parameter under 3-categories and 4-categories did not show noticeable difference under the same calibration sample size, and they were noticeably smaller than those under 2-categories and 5-categories, while the mean(b) of *b*-parameters under 3-categories and 5-categories had similar RMSE values under the same calibration sample size, and they were smaller than those under 2-categories and 4-categories.

It can be seen from Table 4 and Figures 4, 5, the bias of new items had the same trend as the RMSE, The smaller the value of RMSE, the closer the value of bias was to 0.

## Empirical Study

In this paper, an online calibration method based on GRM is proposed, which has a good performance in simulation study. What is the performance on real data? Because the construction of the real CAT item pool is expensive, it is difficult to organize and arrange large-scale CAT tests also. This study used the response data of 500 examinees on 10 polytomous items (3-categories) in HSK4 (Chinese proficiency test) to conduct an empirical study. Detailed steps are as follows.

**Step 1:** 500 examinees were randomly divided into two parts. One was the training set, including the response data of 300 examinees. The other was the testing set, including the response data of 200 examinees.

**Step 2:** The ability parameters of examinees and item parameters are estimated through the training set, then the estimated item parameters are taken as the true parameters.

**Step 3:** For the testing set, the K-fold cross validation method (Tan et al., 2014) is used to simulate and generate the operational items and new items in CAT. In this study, leave-one-out approach was used, that is, each test chose one as new item, and the remaining nine items were as operation items.

**Step 4:** According to the responses of 200 examinees on 9 operational items and the true values of the corresponding item parameters, the ability values of 200 examinees were estimated.

**Step 5:** According to the examinee's ability values obtained in step 4 and their responses to the new item, the parameters of the new item were estimated by the new method proposed in this study.

**Step 6**: Each time a different item was selected as the new item, and then the work in step 3~5 was repeated so that the estimated parameters of each new item could be obtained. Then the RMSE between the estimated parameters and the true parameters were calculated.

Because of the limited real data, this study only analyzed the calibrated sample of 200. The results of the analysis were as follows:*RMSE*_*a*_ = 0.4067, *RMSE*_*b*1_ = 0.4778, *RMSE*_*b*2_ = 0.3218, *RMSE*_*b*3_ = 0.3029.

## Discussion and Future Directions

This research extended OEM and MEM to GRM for online calibration, detailed description of algorithms were given in the article. While online calibration is a complex process, there are many factors affecting the calibration accuracy. In order to make online calibration efficient and practicable under GRM, various factors should be explored clearly. Two simulation studies were conducted to investigate the calibration results under various conditions. The results showed: (1) both OEM and MEM were able to generate reasonably new item parameters with 700 examinees per item, and each has its own merits. (2) The Poly-Sq-Ini method had better performance than Poly-Ini method under most experimental conditions. (3) Compared to the random calibration design, the adaptive calibration design do not improve the calibration accuracy in most conditions. (4) The calibration sample size had an effect on the calibration accuracy. In most conditions, the calibration accuracy increases with the increase of sample size. (5) The number of categories of new items also affected the calibration results, the calibration accuracy of 3-categories items was higher than that of 2-categories, and so on.

In addition, a supplementary study was conducted to investigate the calibration accuracy of GRM online calibration under different CAT scenarios. Eight CAT scenarios, which were fully crossed by sample sizes (2,000 and 3,000) and test lengths (variable-length, fixed-length with 10, 20, and 30 respectively), were investigated. The ability estimation results of CAT and the calibration results of new items under various CAT scenarios were listed in Tables A1–A3. As can be seen from Table A1, for the fixed-length CAT, the estimation accuracy of ability increased with the increase of test length under the same sample size. The RMSE value of variable-length CAT was close to that of test length 10 in fixed-length CAT, which indicated that the test length was about 10 under specified cumulative information. All ability bias values in all CAT scenarios were very close to 0. It showed that the simulated CAT can provide accurate ability estimates for the examinees. As can be seen from Tables A2, A3, (1) the calibration accuracy was acceptable in various CAT scenarios, which showed the robustness of online calibration method under GRM. (2) The estimation accuracy of ability had an effect on the calibration accuracy, but the effect was not monotonous, and there was fluctuation. (3) Under two different sample sizes, the calibration accuracy is higher when the test length is 20.

Several future directions for research can be identified. First, in this paper, the *b*-parameters are randomly selected from the normal distribution and then sort in ascending. The true values of *b*-parameters of new items are random, the following scenarios are possible, such as the *b*-parameters under all categories of an item are less than 0, or are greater than 0, and the difference between adjacent categories is very large or so small. Different scenarios may lead to different calibration results, online calibration based on deliberately designed true parameters of new items is the next research content.

Second, in this paper, only the match*-b* method is considered in the adaptive design, other adaptive design methods are not discussed. There are some adaptive calibration design that practicable and perform well under dichotomously scored models (He and Chen, 2019; He et al., 2019). How to extend these adaptive designs to GRM, and whether it will get the same conclusion as dichotomously scored models are the directions of future research.

Third, the number of categories discussed in this paper was up to 5, which means that the new items can be 2, 3, 4, and 5 categories. If there are more than 5-categories items, whether the new online calibration method is still valid is worthy of further study.

Fourth, there is an interesting phenomenon in the bias of the 5-categories condition. The lower *b*-parameters (*b*1, *b*2) have negative bias, and the higher *b*-parameters (*b*4, *b*5) have positive bias. Does it have anything to do with the calibration methods. Other calibration methods will be extended to GRM in further studies, and observe whether similar phenomenon will also occur. So as to investigate whether the phenomenon It is related to the calibration method, whether it is related to the number of categories of new items, or other factors.

## Data Availability Statement

The raw data supporting the conclusions of this article will be made available by the authors, without undue reservation, to any qualified researcher.

## Author Contributions

SD, ZL, and JX designed experiments. JX and FL carried out experiments. JX analyzed experimental results and wrote the manuscript.

### Conflict of Interest

The authors declare that the research was conducted in the absence of any commercial or financial relationships that could be construed as a potential conflict of interest.

## Appendix

**Table A1:** Estimation accuracy of ability under different CAT scenarios.

**Sample size**	**Test length**	**RMSE**	**Bias**
2,000	Variable-length	0.1904	0.0007
	10	0.1924	−0.0004
	20	0.1340	−0.0008
	30	0.1105	−0.0012
3,000	Variable-length	0.1882	0.0033
	10	0.2012	−0.0001
	20	0.1286	−0.0024
	30	0.1057	0.0050

*For variable-length CAT, the cumulative information was set to 25*.

**Table A2:** RMSE of new item parameters under different CAT scenarios.

**Sample size**	**Test length**	**RMSE**			
		***a***	***b1***	***b2***	***b3***
2,000	Variable-length	0.2483	0.2109	0.1802	0.2294
	10	0.2345	0.2224	0.1557	0.2182
	20	0.2169	0.1954	0.1545	0.2242
	30	0.2232	0.2060	0.1685	0.2357
3,000	Variable-length	0.2337	0.1921	0.1620	0.2203
	10	0.2302	0.2571	0.1668	0.2143
	20	0.2121	0.2102	0.1640	0.2078
	30	0.2069	0.2012	0.1664	0.2235

**Table A3:** Bias of new item parameters under different CAT scenarios.

**Sample size**	**Test length**	**Bias**			
		***a***	***b1***	***b2***	***b3***
2,000	Variable-length	0.0998	−0.0005	−0.0330	−0.0685
	10	−0.0719	−0.0889	−0.0088	0.0615
	20	−0.0001	−0.0464	−0.0011	0.0272
	30	0.0589	−0.0168	−0.0211	−0.0269
3,000	Variable-length	0.0939	−0.0117	−0.0239	−0.0458
	10	−0.0650	−0.1364	−0.0486	0.0287
	20	−0.0228	−0.0187	−0.0106	0.0011
	30	0.0613	−0.0152	−0.0232	−0.0486

*Taking 3-categories items as example and the calibration sample size is set to 500*.
